# Effects of *Lactococcus lactis* on the Intestinal Functions in Weaning Piglets

**DOI:** 10.3389/fnut.2021.713256

**Published:** 2021-08-19

**Authors:** Dongming Yu, Yaoyao Xia, Liangpeng Ge, Bie Tan, Shuai Chen

**Affiliations:** ^1^Guangdong Laboratory of Lingnan Modern Agriculture, College of Animal Science, South China Agricultural University, Guangzhou, China; ^2^Chongqing Academy of Animal Sciences, Chongqing, China; ^3^Laboratory of Animal Nutritional Physiology and Metabolic Process, Key Laboratory of Agro-Ecological Processes in Subtropical Region, Changsha, China; ^4^National Engineering Laboratory for Pollution Control and Waste Utilization in Livestock and Poultry Production, Changsha, China; ^5^Institute of Subtropical Agriculture, Chinese Academy of Sciences, Changsha, China

**Keywords:** *Lactococcus lactis*, amino acid, weaning piglet, intestinal immunity, gut microbiota

## Abstract

Post-weaning diarrhea of piglets is associated with gut microbiota dysbiosis and intestinal pathogen infection. Recent studies have shown that *Lactococcus lactis* (*L.lactis*) could help suppress pathogen infection. This study aimed to investigate the effects of *L.lactis* on various factors related to growth and immunity in weaning piglets. The results showed that *L.lactis* improved the growth performance, regulated the amino acid profile (for example, increasing serum tryptophan and ileal mucosal cystine) and the intestinal GABAergic system (including inhibiting ileal gene expression of SLC6A13, GABAAρ1, π, θ, and γ1, and promoting ileal GABAAα5 expression). *L.lactis* also modulated intestinal immunity by promoting jejunal interleukin 17, 18, 22, ileal toll-like receptor 2, 5, 6, and myeloid differentiation primary response protein 88 gene expression while inhibiting jejunal interferon-γ and ileal interleukin 22 expressions. *L.lactis* highly affected the intestinal microbiota by improving the beta diversity of gut microbiota and the relative abundance of *Halomonas* and *Shewanella*. In conclusion, *L.lactis* improved the growth performance and regulated amino acid profiles, intestinal immunity and microbiota in weaning piglets.

## Introduction

Weaning is the most critical phase in pig production and is generally associated with intestinal infections and diarrhea (1). The biggest challenge for weaning piglets is diarrhea caused by weaning stress and pathogen infection such as enterotoxigenic *Escherichia coli* (ETEC). As an active player in host physiological activities, the gut microbiota plays a vital role in modulating pathogen infection and diarrhea in piglets (2). Weaning changes the gut microbiota in humans, piglets, and cows (3–5), which can result in immune system less development, insufficiency of physiological function (6), and increased risk of pathogen infection (7). Thus, appropriate strategies in microbiology could be used to relieve the stress of weaning and prevent infections.

In the past, antibiotics were wildly used as feed additives to promote growth and prevent pathogens in animal production and disease treatment (8–10). However, the overuse of antibiotics resulted in serious public health problems, such as antibiotic resistance gene transfer and an increase in antibiotic-resistance bacteria. Thus, animal producers in many countries have reduced or eliminated the use of antibiotics in feed (11).

There is a great opportunity to develop new strategies for preventing intestinal pathogen infection in weaning piglets. Probiotics can prevent infections caused by pathogens such as *Clostridium difficile* (12, 13). However, the ability of probiotics to prevent infection varies (14). *Lactococcus lactis* (*L.lactis*) was recently reported to prevent cholera (15, 16). Our previous research showed that *L.lactis* regulated the intestinal immune reaction via gamma-aminobutyric acid (GABA) production and prevented pathogen infections in piglets (17, 18). These findings suggest that *L.lactis* has great potential to prevent intestinal infections in piglets.

The current study aimed to evaluate the modulatory role of *L.lactis* in growth performance, amino acid profile, intestinal immunity, and gut microbiota in piglets.

## Materials and Methods

### Animals and Experiment Design

Fifteen healthy piglets (Duroc × Landrace × Landrace, aged 21 days) were purchased from Hunan New Wellful Co., Ltd (Changsha, China). After an adaption period of 3 days, piglets were randomly assigned to the control group (*n* = 7) and the *L.lactis* group (*n* = 8). This study shared the data of the control group with our previous research (19). The piglets in the *L.lactis* group were orally dosed with *L.lactis* (2.0^*^10^9^ CFU/ml, 20 ml) on days 1 and 8. All piglets were fed a corn-and soybean meal-based diet (Supplementary Table 1), and other feedings and environmental control conditions were the same as in our previous study (19). Body weight and feed intake were monitored weekly throughout the experiment, and average daily gain (ADG), average daily feed intake (ADFI), and feed conversion ratio (FCR) were calculated. At the end of week 3, piglets were sacrificed after anesthesia.

The blood, jejunum, jejunal mucosa, ileum, ileal mucosa, colon and luminal content were collected immediately, snap-frozen in liquid nitrogen, and stored at −80°C until further processing. All animal experiment procedures were approved by the Animal Welfare Committee of the Institute of Subtropical Agriculture, Chinese Academy of Sciences (2016-4B).

### Culture of *L.lactis*

*L.lactis* (ATCC 19435) was grown overnight in 5 ml of M17 medium (Thermo Fisher Scientific, Waltham, MA USA) broth at 37°C with gentle agitation (180 rpm/min). The next day, 3 ml of M17 medium was inoculated with 100 μl of the overnight culture for further amplification and culture.

### Diarrhea Index and Counting of *E.coli*

The diarrhea index and diarrhea rate data of piglets were recorded daily according to the criterion of feces score (Supplementary Table 2). The *E.coli* loads in the jejunal mucosa, ileal mucosa, and colonic content were quantified by Maconkey Agar (Sigma-Aldrich, Burlington, United States) according to the previous work (17).

### Free Amino Acids Analysis

According to our previous report (19), the ileal mucosa and serum amino acid levels were measured using high-performance liquid chromatography. Authentic standards (Sigma-Aldrich, Burlington, United States) were used to quantify the amino acids in the samples.

### Gene Expression Analysis

Expression of the GABAergic system and immune-associated genes was analyzed by reverse transcriptase-polymerase chain reaction (RT-PCR), and primers (Supplementary Table 3) were selected according to our previous study (19). The samples were individually normalized to the housekeeping genes, β-actin (ACTB) and glyceraldehyde-3 phosphate dehydrogenase (GAPDH). The relative gene expression was calculated by formula 2^−(ΔΔCT)^.

### 16S rDNA Sequencing With Illumina MiSeq Sequencing

We used 16S rDNA gene sequencing to analyze the V3–V4 region of ileal microbiota according to our previous study (19). The QIAGEN QIAamp DNA Stool Mini Kit (Qiagen, Hilden, NRW, Germany) was used to extract DNA from the ileal contents and Agarose gel electrophoresis was used to quantify the DNA. Sequencing libraries were then generated using the Ion Plus Fragment Library Kit (Thermo Fisher Scientific, Waltham, MA, USA), assessed on the Qubit® 2.0 Fluorometer (Thermo Fisher Scientific, Waltham, MA, USA), and sequenced on the Illumina MiSeq Sequencer. Under specific filtering conditions, the raw data were filtered to obtain high-quality clean reads according to the Cutadapt quality control process. Uparse software (Uparse v7.0.1001) was used for sequence analysis and operational taxonomic unit (OTU) clustering and the identity threshold was set to 97%. The species annotation was performed with the RDP Classifier (V2.2, Michigan State 14 University Board of Trustees, East Lansing MI) based on the GreenGene database. MUSCLE software (Version 3.8.31) was used for phylogenetic relationship analysis. Subsequently, we used R and QIIME software (V 1.7) on the normalized output data to analyze the alpha diversity, beta diversity, and environmental factor correlation (Spearman analysis). The FAPROTAX database was used for function prediction. Illumina MiSeq sequencing, processing of sequencing data, and bioinformatics analysis were performed by Beijing Novogene Bioinformatics Technology Co., Ltd. (Beijing, China).

### Environmental Factor Correlation Analysis

According to the relative abundance at the phylum level, the top 28 taxa were used for correlation analysis with growth performance indicators, amino acid profiles, and intestinal immune factors.

### Statistical Analyses

The results were expressed as the mean ± standard error of the mean (SEM). All data were pre-processed with Excel 2019 (Microsoft, Redmond, Washington, USA). Word 2019 software (Microsoft, Redmond, Washington, USA) was used to prepare tables, and GraphPad Prism 8.0 (GraphPad Software, Inc., La Jolla, CA, USA) was used to analyze statistics and generate figures. If the data followed a normal distribution, an unpaired *t*-test was used for the statistical analysis between the two groups; otherwise, the Wilcoxon signed-rank test was used for analysis. A *P*-value < 0.05 was considered statistically significant.

## Results

### *L.lactis* Partly Improved the Growth Performance of Weaning Piglets

**Figure 1:**
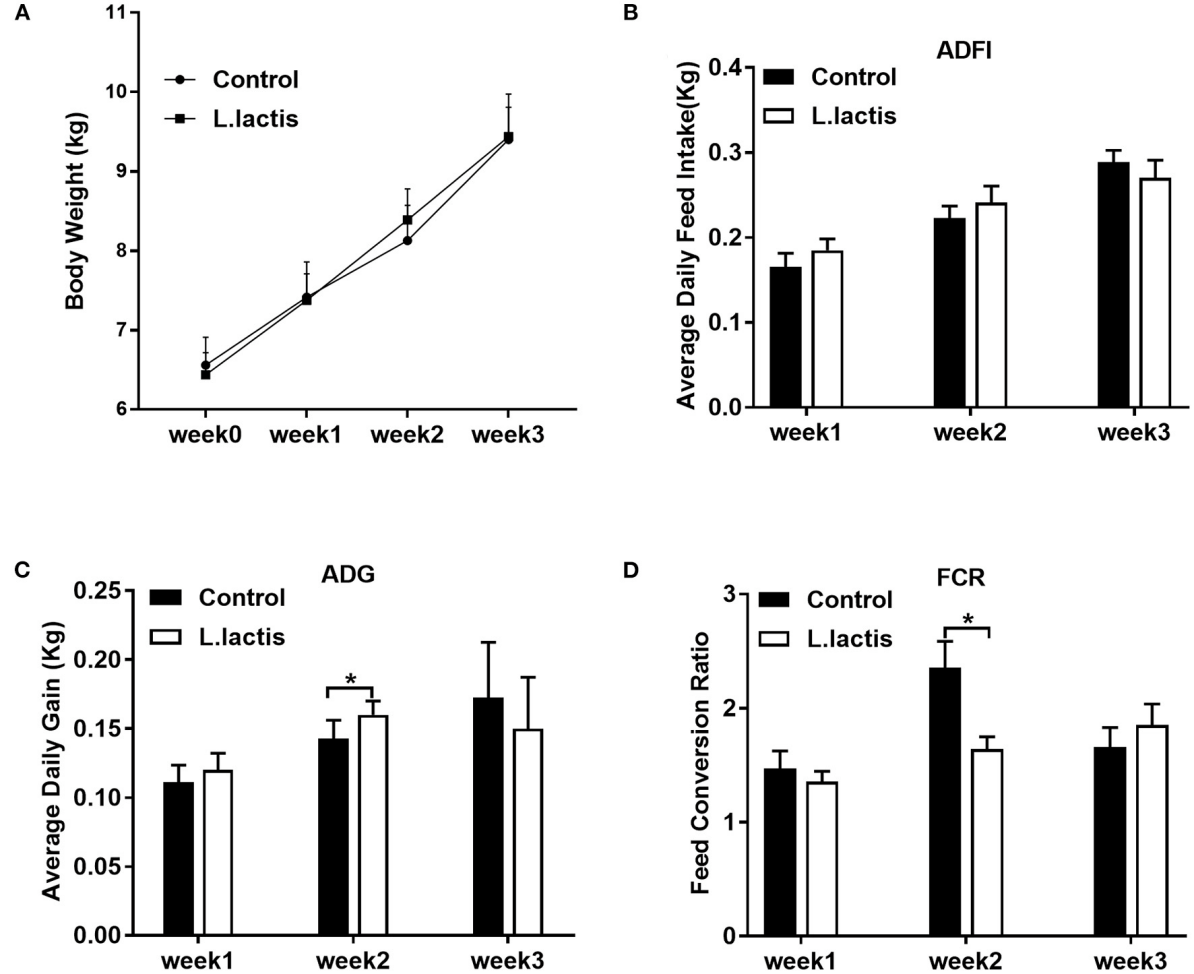
Effects of *L.lactis* on piglet growth performance. **(A)** Body weight; **(B)** average daily feed intake; **(C)** average daily gain; **(D)** feed conversion ratio (FCR). An unpaired *t*-test was used for analyzing the data (mean ± SEM). *P < 0.05.

The body weight and average daily feed intake of piglets were similar between the control and *L.lactis* groups (Figures 1A,B). *L.lactis* increased average daily weight gain and reduced FCR in the 2nd week (*P* < 0.05), while did not affect them in the other 2 weeks (Figures 1C,D).

### *L.lactis* Reduced Intestinal *E.coli* Load

**Figure 2:**
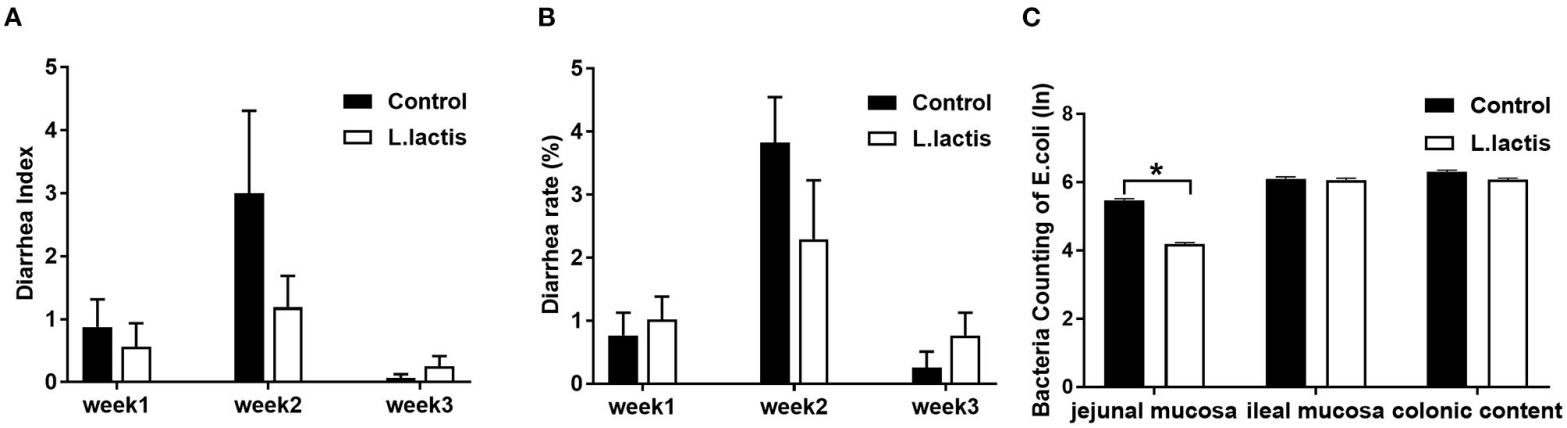
Effects of *L.lactis* on diarrhea and counting of *E.coli*. **(A)** Diarrhea index; **(B)** diarrhea rate; **(C)** *E.coli* count. Wilcoxon rank-sum test was used to analyze the data (mean ± SEM). *P < 0.05.

Results of diarrhea index and diarrhea rate showed that *L.lactis* did not affect the diarrhea of piglets (Figures 2A,B). And *L.lactis* reduced *E.coli* load (*P* < 0.05) in jejunal mucosa but not ileal mucosa and colonic content (Figure 2C).

### *L.lactis* Regulated the Amino Acid Profiles

*L.lactis* significantly increased (*P* < 0.05) the concentrations of L-cystine and decreased (*P* < 0.05) the level of L-glutamic acid in ileal mucosa (Table 1). In peripheral circulation, the serum level of L-tryptophan (Trp) was improved (*P* < 0.05) due to *L.lactis* administration (Table 1).

**Table 1:** Effects of *L.lactis* on the ileal mucosa (μg/g) and serum (μg/mL) the amino acid profiles.

	**Control**	***L.lactis***	***P*-value**
**Ileal mucosa**			
L-Alanine	283.96 ± 5.83	280.49 ± 5.53	0.71
L-Valine	92.78 ± 8.77	98.29 ± 2.58	0.60
L-Leucine	224.37 ± 14.95	214.48 ± 3.48	0.61
L-Isoleucine	108.68 ± 7.88	101.49 ± 4.84	0.47
L-Phenylalanine	132.41 ± 8.30	134.52 ± 1.23	0.84
L-Tryptophan	21.46 ± 1.57	20.54 ± 0.67	0.64
L-Methionine	88.81 ± 7.49	85.77 ± 1.54	0.75
L-Proline	136.35 ± 10.11	135.88 ± 2.66	0.96
Glycine	509.36 ± 44.67	446.92 ± 12.5	0.26
L-Serine	179.35 ± 11.26	187.20 ± 6.51	0.58
L-Threonine	97.75 ± 10.06	92.06 ± 4.17	0.63
L-Cystine	24.24 ± 1.93	37.32 ± 1.69^*^	0.01
L-Tyrosine	125.17 ± 9.13	128.06 ± 5.54	0.80
L-Aspartic acid	242.80 ± 14.60	214.95 ± 14.17	0.22
L-Glutamic acid	1183.57 ± 52.8	997.92 ± 30.01^*^	0.01
L-Lysine	148.06 ± 11.61	150.80 ± 2.56	0.84
L-Arginine	131.12 ± 11.43	134.16 ± 2.29	0.82
L-Histidine	45.74 ± 1.93	47.87 ± 1.15	0.40
**Serum**			
L-Histidine	7.00 ± 0.53	8.57 ± 0.56	0.09
L-Serine	16.87 ± 0.18	18.00 ± 1.85	0.60
L-Arginine	20.92 ± 0.65	18.34 ± 2.22	0.34
Glycine	42.92 ± 4.10	48.72 ± 6.79	0.50
L-Aspartic acid	4.29 ± 0.48	4.53 ± 0.57	0.76
L-Glutamic acid	42.33 ± 1.63	48.21 ± 3.47	0.17
L-Threonine	5.40 ± 0.10	5.27 ± 0.38	0.78
L-Alanine	42.84 ± 1.97	43.62 ± 5.86	0.90
L-Proline	30.17 ± 1.18	30.79 ± 3.40	0.87
L-Cystine	2.01 ± 0.35	2.31 ± 0.26	0.55
L-Lysine	42.92 ± 3.12	38.49 ± 2.34	0.30
L-Tyrosine	19.55 ± 1.44	18.48 ± 0.70	0.54
L-Methionine	9.49 ± 0.79	7.46 ± 0.75	0.10
L-Valine	19.41 ± 1.24	20.51 ± 1.92	0.67
L-Isoleucine	12.11 ± 0.57	11.59 ± 0.39	0.51
L-Leucine	18.00 ± 0.71	19.04 ± 2.33	0.71
L-Phenylalanine	12.71 ± 0.94	14.28 ± 1.32	0.38
L-Tryptophan	4.75 ± 0.18	6.56 ± 0.62^*^	0.02

*Amino acid profiles of the ileal mucosa and serum were detected by HPLC. Piglets from control group (n = 7) and L.lactis group (n = 8). Unpaired t-test was used to analyze data (mean ± SEM)*.

* *P < 0.05*.

### *L.lactis* Affected Intestinal Immunity

**Figure 3:**
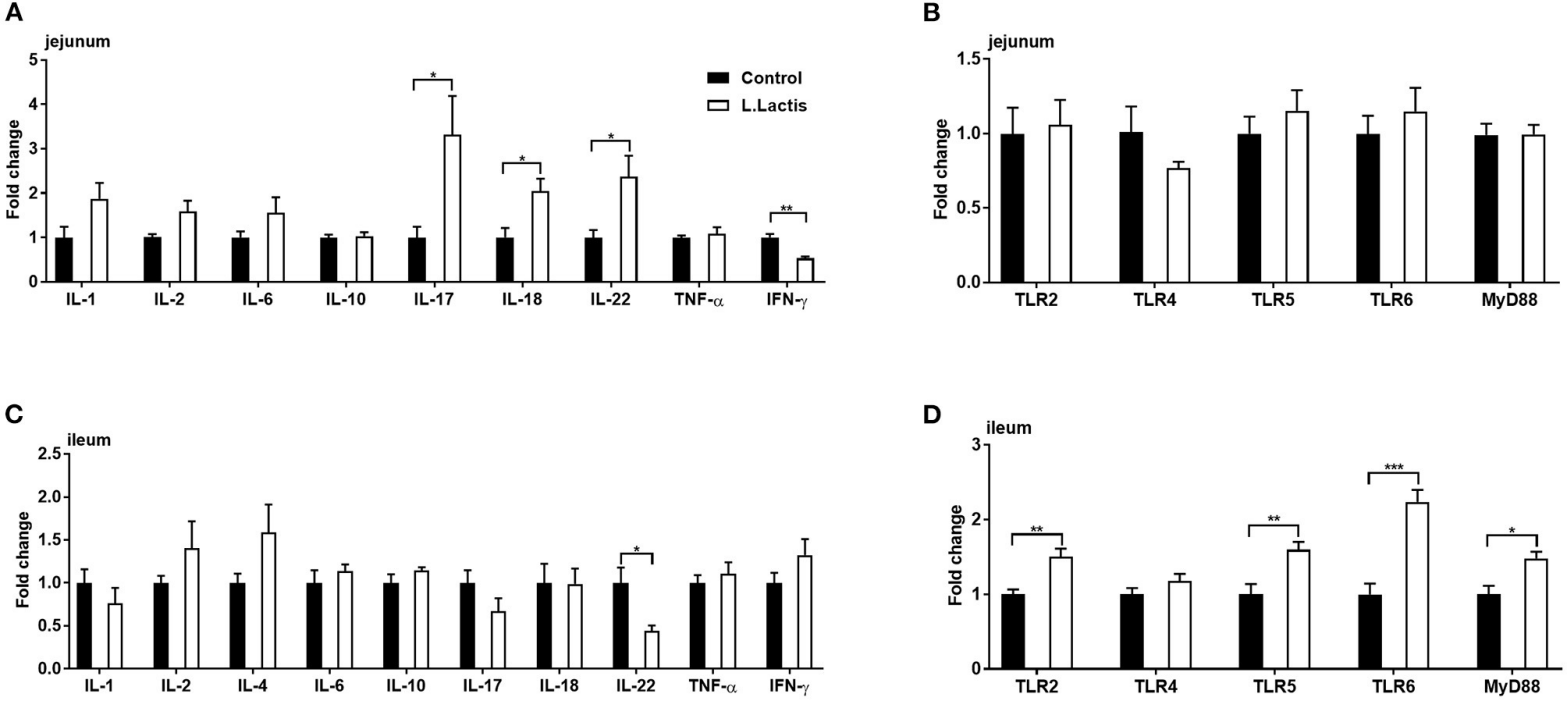
Jejunal and ileal mRNA expression of immune-related factors. Relative gene expression of **(A,C)** IL-1, 2, 4, 6, 8, 10, 17, 18, 22, TNF-α, and IFN-γ, and **(B,D)** TLR-2, 4, 5, 6, and MyD88 were analyzed by RT-PCR (jejunal IL-4 was undetected). An unpaired *t*-test was used for analyzing the data (mean ± SEM). *P < 0.05; **P < 0.01; ***P < 0.001.

To examine the effect of *L.lactis* on intestinal immunity, we used RT-PCR to measure the mRNA expression of jejunal and ileal immunity-related factors, including toll-like receptors (TLR)-2, 4, 5, and 6, myeloid differentiation primary response protein-88 (MyD88), tumor necrosis factor-alpha (TNF-α), interferon-gamma (IFN-γ), and interleukin (IL)-1, 2, 4, 6, 10, 17, 18, and 22. In the *L.lactis* group, jejunal IFN-γ (*P* < 0.01) and ileal IL-22 (*P* < 0.05) were reduced, and jejunal IL-17 (*P* < 0.05), 18 (*P* < 0.05), and 22 (*P* < 0.05), ileal TLR-2, 5, 6 (*P* < 0.01), and MyD88 (*P* < 0.05) were increased, while other factors was not changed, comparing with the controls (Figure 3).

### *L.lactis* Regulated the Intestinal GABAergic System

The mRNA expression of the gut GABAergic system was analyzed using RT-PCR. The results showed that the expression of SLC6A13 was inhibited (*P* < 0.05) due to *L.lactis* treatment (Table 2). Analysis of the gene expressions of GABA receptors (GABAB1-2, GABAAα1-5, β2, γ1-2, δ, ε, π, θ, and ρ1) showed that *L.lactis* inhibited the expression of GABAAρ1, π, θ, and γ1 (*P* < 0.05), while it increased GABAAα5 expression (*P* < 0.05) (Table 2).

**Table 2:** Expression of GABAergic system in the ilea of piglets.

	**Control**	***L.lactis***	***P*-value**
SLC6A1	1.00 ± 0.18	1.21 ± 0.09	0.66
SLC6A11	1.00 ± 0.09	0.68 ± 0.14	0.09
SLC6A12	1.00 ± 0.04	0.88 ± 0.11	0.79
SLC6A13	1.00 ± 0.12	0.61 ± 0.05^*^	0.01
GABAB1	1.00 ± 0.10	0.88 ± 0.07	0.27
GABAB2	1.00 ± 0.17	0.95 ± 0.20	0.98
GABAAβ2	1.00 ± 0.12	1.09 ± 0.29	0.69
GABAAδ	1.00 ± 0.12	0.89 ± 0.12	0.38
GABAAε	1.00 ± 0.25	0.79 ± 0.11	0.20
GABAAρ1	1.00 ± 0.25	0.55 ± 0.06^*^	0.04
GABAAπ	1.00 ± 0.13	0.24 ± 0.03^*^	0.01
GABAAθ	1.00 ± 0.13	0.30 ± 0.04^*^	0.01
GABAAγ1	1.00 ± 0.16	0.46 ± 0.05^*^	0.01
GABAAγ2	1.00 ± 0.12	0.93 ± 0.16	0.57
GABAAα1	1.00 ± 0.02	0.92 ± 0.09	0.48
GABAAα2	1.00 ± 0.39	1.60 ± 0.52	0.79
GABAAα3	1.00 ± 0.13	0.80 ± 0.06	0.15
GABAAα4	1.00 ± 0.17	1.44 ± 0.12	0.18
GABAAα5	1.00 ± 0.08	1.64 ± 0.12^*^	0.01

*Ileal gene expression of GAT, GABA receptors, and GAD (undetected) were analyzed by RT-PCR. Piglets from control group (n = 7) and L.lactis group (n = 8). Unpaired t-test was used to analyze the data (mean ± SEM)*.

* *P < 0.05*.

### *L.lactis* Shifted the Gut Microbiota

**Figure 4:**
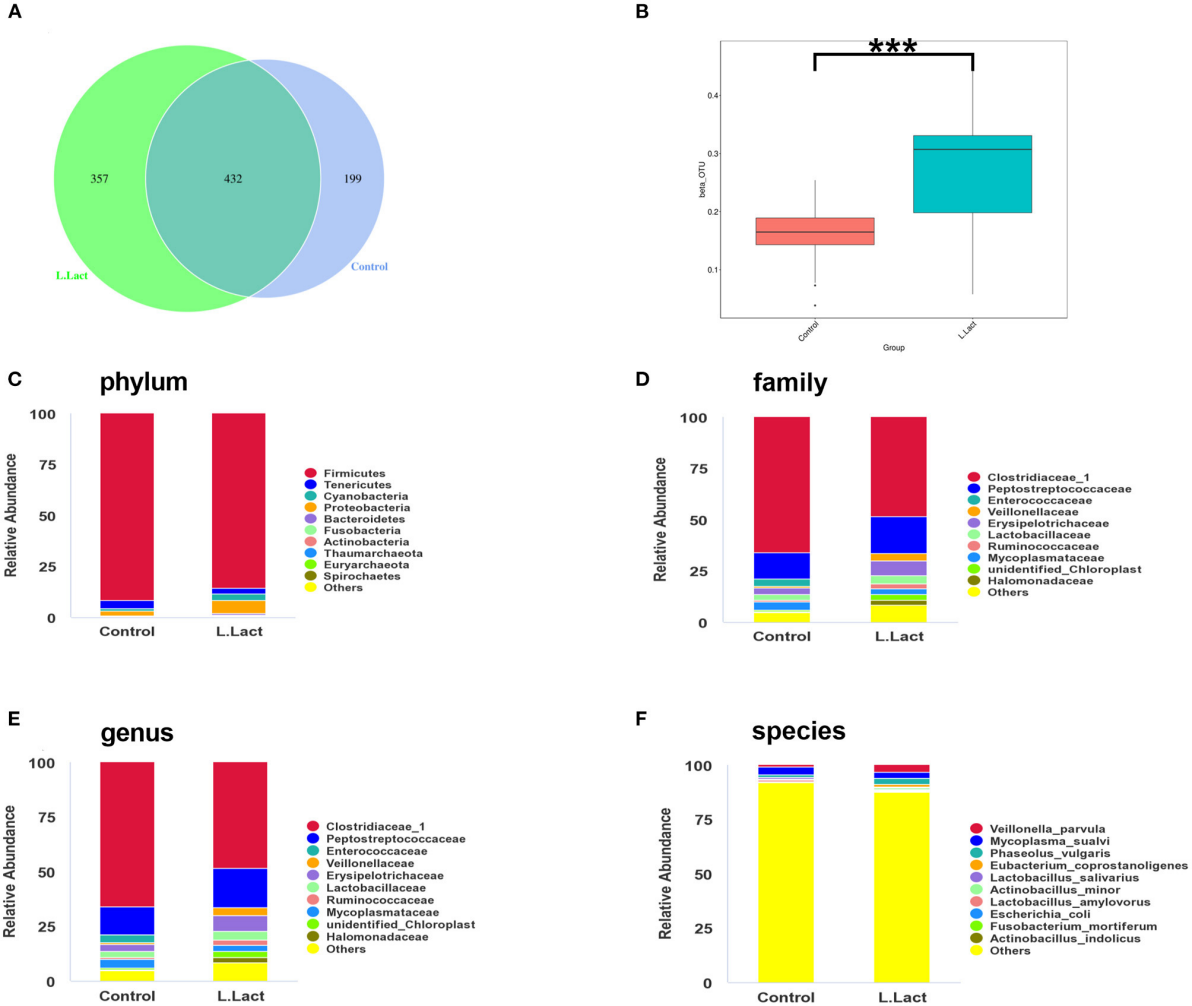
Effects of *L.lactis* on ileal microbiota of the piglets. **(A)** The Venn diagram shows the common and unique OTUs of the control and *L.lactis* groups. **(B)** Rank abundance curves of beta diversity in the control and *L.lactis* groups. **(C–F)** Relative abundance of top 10 phyla **(C)**, families **(D)**, genera **(E)**, and species **(F)** in the control and *L.lactis* groups. ***P < 0.001.

**Figure 5:**
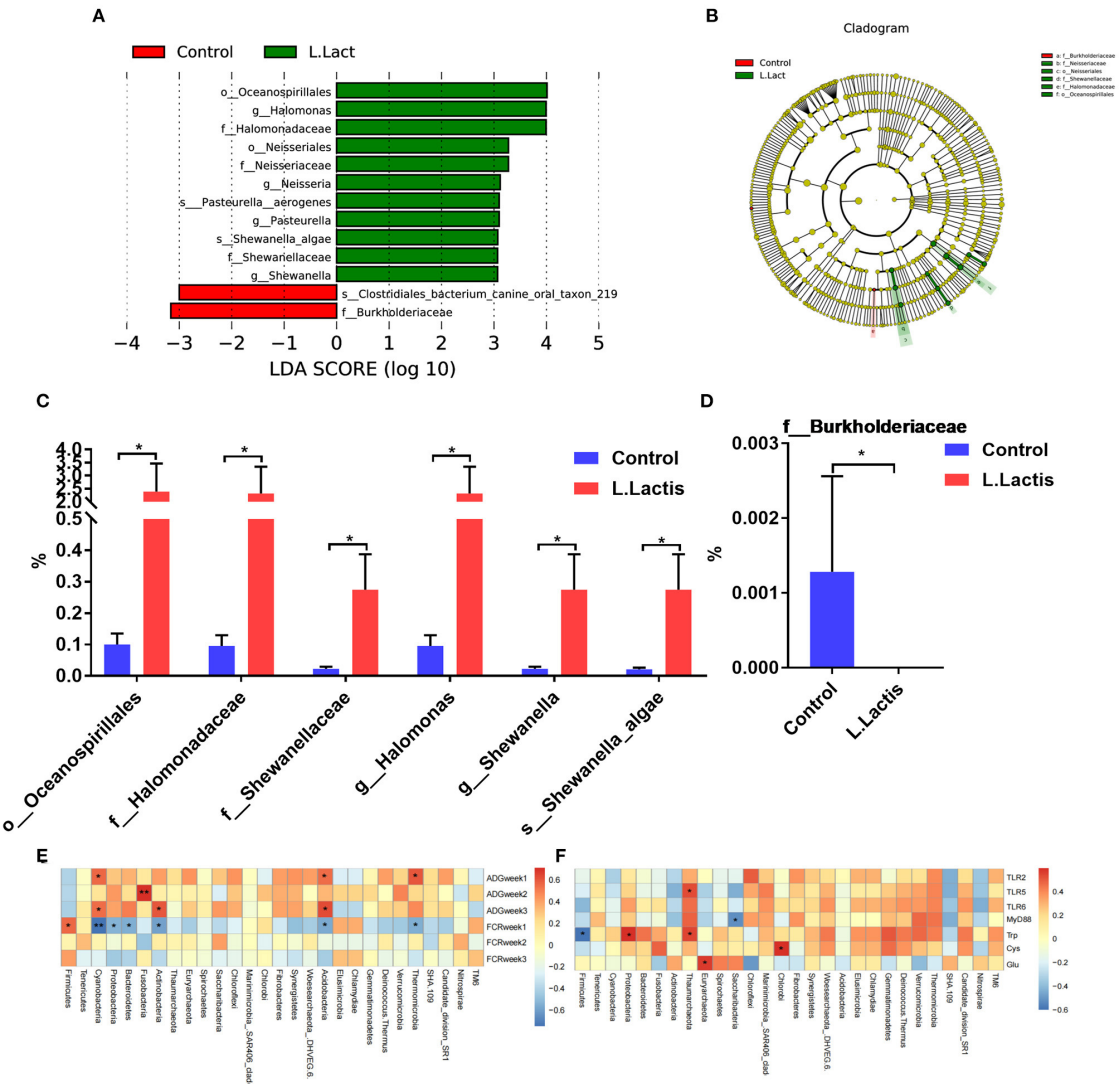
*L.lactis* regulated the ileal microbiota of the piglets. **(A–D)** LEfSe analysis demonstrated the significant members in the control and *L.lactis* groups. **(E)** Environmental factor correlation analysis indicated that ADG of week 2 was positively correlated with Fusobacteria. **(F)** Ileal TLR-5 mRNA expression and serum tryptophan (Trp) level were positively correlated with the relative abundance of *Thaumarchaeota*; serum Trp level was also positively correlated with the relative abundance of *Proteobacteria*. *P < 0.05; **P < 0.01.

The ileal microbiota was analyzed by 16S rDNA sequencing. According to the Venn diagram, 988 OTUs were clustered, in which 199 and 357 OTUs were unique in the control and *L.lactis* group, separately (Figure 4A). The Beta diversity analysis showed a remarkable difference between control and *L.lactis* groups (Figure 4B), while the Alpha diversity analysis showed no difference (Supplementary Table 4). At the phylum, family, genus, and species level, *Firmicutes, Clostridiaceae_1, Clostridium_sensu_stricto_1*, and *Veillonella parvula* were by far the dominative populations (Figures 4C–F). According to Linear discriminant analysis effect size (LEfSe) results, *Oceanospirillales, Halomonas*, and *Halomonadaceae* were enriched in the *L.lactis* group, while *Burkholderiaceae* and *Clostridiales bacterium_canine_oral_taxon_219* were enriched in the controls (Figures 5A,B). *L.lactis* increased the relative abundance of *Oceanospirillales, Halomonadaceae, Shewanellaceae, Halomonas, Shewanella*, and *Shewanella_algae* (Figure 5C), and reduced the relative abundance of *Burkholderiaceae* (Figure 5D). Spearman correlation analysis indicated that ADG of the 2nd week was positively correlated with the relative abundance of *Fusobacteria* (Figure 5E). Ileal TLR-5 mRNA expression and the level of Trp in serum were positively correlated with the relative abundance of *Thaumarchaeota*. The serum level of Trp also was positively correlated with the relative abundance of *Proteobacteria* (Figure 5F).

## Discussion

The biggest challenge that weaning piglets faced is diarrhea caused by weaning stress and pathogen infection. During weaning, the gut microbiota of piglets is maladjusted due to diet and environmental changes (1), often leading to infection (2). Previous studies showed that the administration of probiotics could reduce weaning stress and pathogen infection by regulating the gut microbiota (20, 21). Our study showed that *L.lactis* improved growth performance and modulated intestinal immunity, ileal microbiota, and amino acid profiles of ileal mucosal and serum in weaning piglets.

Weaning stress impairs the feed intake and growth performance of the weaned piglets. Our previous research (18) showed that *L.lactis* promoted intestinal GABA production, and GABA was reported to enhance the growth performance (19) and inhibit the expression of cholecystokinin-related genes (22). We found that *L.lactis* partly increased the ADG and reduced the FCR in the 2nd week. The mechanism might be that GABA produced by *L.lactis* increased the secretion of hormones closely related to growth performance. The ADG and FCR of week 3 were similar between the control and *L.lactis* groups. The possible reason is that the *L.lactis* transplantation has a time-limited effect on piglets. Our results contradicted a previous finding that the administration of *L.lactis* reduced body weight (23). The difference may be explained by different animal models or different dosages of *L.lactis*. However, the mechanism under the improvement of growth performance driven by *L.lactis* needs to be further studied.

The weaning stress of piglets usually causes diarrhea, slowing down the growth of piglets. Weaning piglets are susceptible to diarrhea caused by pathogenic *E.coli* (e.g., ETEC) infection. Growing studies indicate that probiotics prevent pathogenic bacteria colonization and proliferation (12, 13). Manuela et al. (24) showed that probiotics inhibited pathogenic bacteria by producing antimicrobial metabolites and competing for energy substances. *Lactic acid bacteria* have been found to produce various antimicrobial substances (e.g., lactic acid, bacteriocins, and hydrogen peroxide) to inhibit pathogenic bacteria colonization (25–27). Similarly, we found that *L.lactis* reduced the jejunal mucosal *E.coli* load, which helps prevent diarrhea caused by harmful *E.coli* colonization.

The GABAergic system plays vital role in intestinal health and disease, partly relying on hormone secretion and intestinal immunity (28, 29). Therefore, we analyzed the effect of *L.lactis* on the intestinal GABAergic system. An increasing number of studies have illustrated the critical roles of GABA transporters (GAT) on health and diseases. Xia et al. (30) showed that GAT2 (SLC6A13) sustained IL-1β production in macrophages, and Ren et al. (18) identified the role of GAT2 in the defense against pathogen infection. This study found that *L.lactis* reduced GAT2 expression and regulated GABA receptors. Thus, *L.lactis* transplantation caused significant regulation of the intestinal GABAergic system. *L.lactis* can regulate the function of immune cells by regulating the intestinal GABAergic system, thus maintaining intestinal homeostasis. However, these results are limited in clarifying the relationship between *L.lactis*, intestinal GABAergic system and intestinal immune responses.

Amino acids play an essential role as reactive substances in peptide and protein biosynthesis. Moreover, recent studies have shown that amino acids (e.g., tryptophan, cysteine) contribute to the metabolic reprogramming of immune cells such as T cells and macrophages (31). For example, tryptophan is required for T cell proliferation and activation, and tryptophan metabolism is enhanced in activated immune cells. Our study showed that *L.lactis* transplantation increased the tryptophan level of serum, which might subsequently activate immune cells to resist pathogenic infection. Cysteine, by facilitating glutathione synthesis, plays a vital role in maintaining redox balance to support the function of immune cells (32). In this study, the improved level of ileal mucosal cysteine facilitated by *L.lactis* might further generate glutathione to counter the production of reactive oxygen species that can cause cell death at high concentrations. Many amino acids such as tryptophan, cysteine and glutamic acid are regulators of growth performance, intestinal immunity, and gut microbiota, indicating that *L.lactis* promotes growth performance and regulates intestinal immunity might partly by affecting amino acids.

The intestinal tract is the primary organ for food digestion and nutrient absorption and is also the largest immune organ. The intestinal immune system is essential to resisting pathogen infection (33). Our study showed that *L.lactis* promoted the ileal expression of TLR-2, 5, and 6, as well as MyD88, in weaning piglets. These TLRs recognize different pathogenic components and activate immune cells to kill pathogens (34). Therefore, *L.lactis* could activate immune cells by activating TLRs signaling pathways, thus resisting intestinal infection. According to our previous research, ETEC-infection increased the abundance of *L.lactis*, promoting the T helper cell 17 (Th17) immune response via GABA production (18). Indeed, *L.lactis*-promoted jejunal IL-17 gene expression was also observed in this study. Our previous study showed that GABA supplementation could increase the expression of intestinal SLC6A13 during ETEC infection (35). Thus, the glutamate in the intestine might be used for GABA production in this study. It was reported that the glutamine-glutamate-GABA metabolic pathway supports the Th17 immune reaction to IL-17 production (36). According to these results, the intestinal GABA derived from host glutamate metabolism and *L.lactis* might support the Th17 immune reaction. Our study also found that *L.lactis* increased the jejunal gene expression of IL-18 and IL-22 and reduced the ileal gene expression of IL-22. IL-18 can induce the intestinal epithelium to produce antimicrobial proteins (37), while, intestinal IL-22 signaling was positively correlated with the differentiation and antimicrobial effect of Paneth cells (38), and IL-22 has been reported to promote intestinal stem cell-mediated epithelial regeneration (39). Thus, *L.lactis* transplantation regulates intestinal immune response, which would help maintain intestinal immune homeostasis. For example, *Lactobacillus* could mitigate colitis by producing aryl hydrocarbon receptor agonists (AHR) (40).

The gut microbiota affects many physiological functions of the host and is linked to the pathogenesis of various diseases such as inflammatory bowel disease (41), cancer (42), and obesity (43). Numerous studies have reported that health and disease markers highly correlate with the gut microbiome (44), and the occurrence of various diseases is associated with the decrease of intestinal microbial diversity (45). Recent studies demonstrated that many probiotics regulated gut microbiota and inhibited intestinal diseases (46). As a promising non-colonizing probiotic, it is reasonable that *L.lacatis* was undetectable after short-term and low dosage administration. Although it did not change the relative abundance of intestinal *L.lactis* after treatment for 2 weeks, *L.lactis* was found sifted and regulated the gut microbiota, such as enriching some beneficial bacteria and suppressing potential pathogenic bacteria. *L.lactis* treatment reduced the relative abundance of *Burkholderia*, which is highly related to inflammatory bowel disease and intestinal infection (47, 48). And *L.lactis* transplantation increased the relative abundance of *Shewanella* which benefits pancreatic beta cell expansion and insulin production (49). Li et al. (50) showed that transplantation of fecal bacteria from healthy pigs improved the growth status of the recipient pigs, although the overall composition of intestinal bacteria could not be changed, some potential probiotics were significantly enriched. Derrien et al. (51) showed that probiotics do not significantly alter the composition of fecal microbiota in healthy adults but can help maintain the dynamic balance of gut microbiota and reduce the adverse effects of intestinal microbial disorders. Therefore, the function of *L.lactis* may be more dependent on maintaining the dynamic balance of gut microbiota and microbial metabolic activities. For example, tryptophan metabolites of gut microbiota can improve intestinal barrier function and alleviate dextran sulfate sodium (DSS)-induced colitis in mice (52). Spearman correlation analysis of our data indicated that the effect of *L.lactis* on the gut microbiota was closely related to amino acid profiles, growth performance, and intestinal immunity. Thus, *L.lactis* may influence the intestinal microbiota to help regulate these factors in weaning piglets.

## Conclusion

*L.lactis* improved the growth performance, regulated amino acid profiles and intestinal immunity in weaning piglets, which might be associated with changing the intestinal microbiota. These results would help evaluate the feasibility of *L.lactis* in pig production to reduce the negative health effects of weaning.

## Data Availability Statement

The datasets presented in this study can be found in online repositories. The names of the repository/repositories and accession number(s) can be found below: 16S rDNA gene profiling data were available in the NCBI database under BioProject PRJNA745933.

## Ethics Statement

The animal study was reviewed and approved by the Animal Welfare Committee of the Institute of Subtropical Agriculture, Chinese Academy of Sciences.

## Author Contributions

SC, BT, and LG designed the experiment and reviewed and revised the manuscript. DY, SC, and YX conducted the experiment. DY and SC analyzed the data. DY and LG prepared tables and figures. DY prepared the manuscript. All authors contributed to the article and approved the submitted version.

## Conflict of Interest

The authors declare that the research was conducted in the absence of any commercial or financial relationships that could be construed as a potential conflict of interest.

## Publisher's Note

All claims expressed in this article are solely those of the authors and do not necessarily represent those of their affiliated organizations, or those of the publisher, the editors and the reviewers. Any product that may be evaluated in this article, or claim that may be made by its manufacturer, is not guaranteed or endorsed by the publisher.

## Abbreviations

*L.lactis*: *Lactococcus lactis*
MyD88: myeloid differentiation primary response protein 88
ADFI: average daily feed intake
ADG: average daily weight gain
*E.coli*: *Escherichia coli*
ETEC: Enterotoxigenic *E.coli*
GABA: gamma-aminobutyric acid
GAD: glutamic acid decarboxylase
GAT: GABA transporter
SLC: solute carriers
IFN-γ: interferences-gamma
IL: interleukin
AHR: aryl hydrocarbon receptor
OTU: operational taxonomic unit
RT-PCR: reverse transcription-polymerase chain reaction
SEM: standard error of the mean
TNF-α: tumor necrosis factor-alpha
DSS: dextran sulfate sodium.
