# Supplementary material for: Effects of Lactococcus lactis on the Intestinal Functions in Weaning Piglets
Source: Front Nutr. 2021 Aug 19;8:713256. doi: 10.3389/fnut.2021.713256 (PMC8416905; doi:10.3389/fnut.2021.713256)
Supplement: Supplementary file 1 [file Data_Sheet_1.docx]

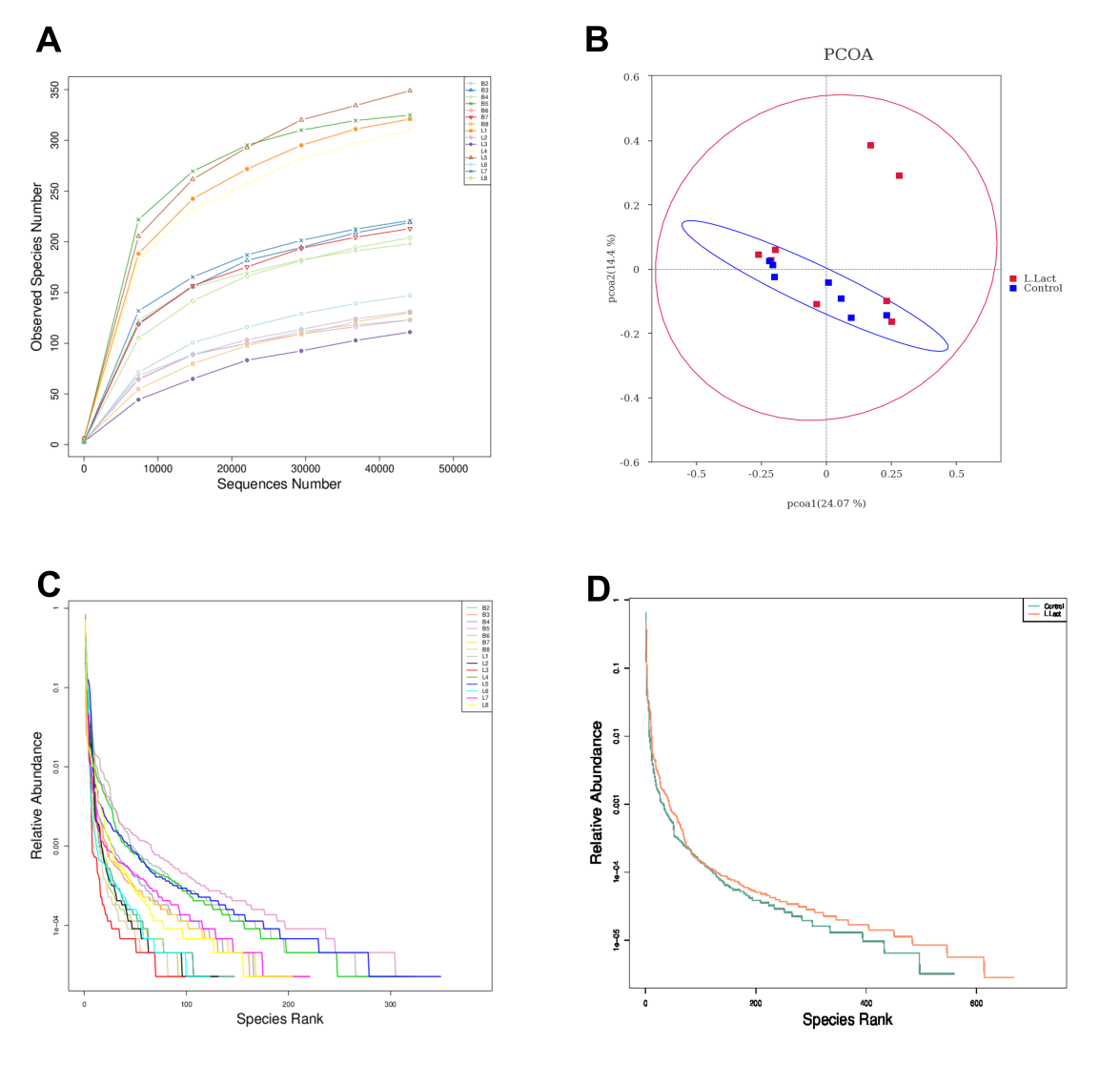


Supplementary Figure 1. The supplementary data of 16srDNA sequencing. (A) Rarefraction curve revealed that sequencing data, sequencing depth and coverage met the demand of the followed analysis. (B) Beta diversity analysis based on PCOA, contrasted the differences between *L.lactis* and control groups. (C,D)Rank abundance curve (C, per sample; D, group) shown that *L.lactis* altered Alpha diversity of gut microbome. control group (n = 7); *L.lactis* group (n = 8).

**Supplementary Table 1** Composition and nutrients contents of the basal diet.

| **Ingredients** | **Basal diet (%)** | **Analyzed chemical composition** |  |
| --- | --- | --- | --- |
| Corn | 61.00 | Dry matter (%) | 85.99 |
| Soybean meal | 10.00 | Crude protein^2^ (%) | 18.89 |
| Extruded soybean | 8.00 | Crude fiber (%) | 2.20 |
| Fermented soybean | 5.00 | Net energy (kcal/kg) | 2330.03 |
| Whey powder | 8.00 | Digestive energy (kcal/kg） | 3467.31 |
| Fish meal | 3.00 |  |  |
| Soybean oil | 0.70 |  |  |
| Sucrose | 1.00 |  |  |
| Premix^1^ | 1.00 |  |  |
| Limestone power | 0.51 |  |  |
| Total | 100.00 |  |  |

^1^ Premix provided the following amounts of vitamins and trace minerals per kg of the complete diet: Organic trace minerals 0.15%; Piglets vitamin 0.04%; Carrier 0.38%; Antioxidant 0.1%; Calcium dihydrogen phosphate 0.78%; Salt 0.37%; Lysine HCL(98%) 0.64%.

^2^ The analyzed composition of amino acids (%, as-fed basis) in the basal diet was as follows: alanine, 0.86; arginine, 1.35; aspartate plus asparagine, 1.87; cysteine, 0.18; glutamate plus glutamine, 3.01; glycine, 0.91; histidine, 0.43; isoleucine, 0.80; leucine,1.47; lysine, 2.02; methionine, 0.21; phenylalanine, 0.87; proline, 0.89; serine, 0.92; threonine, 0.74; tyrosine, 0.47; and valine, 0.79.

**Supplementary Table 2** The criterion of feces score.

|  | Appearance of feces | Diarrhea index |
| --- | --- | --- |
| No diarrhea | Certain shape | 0 |
| Mild diarrhea | Soft feces | 1 |
| Moderate diarrhea | Sticky feces | 2 |
| Severe diarrhea | Liquid feces | 3 |

**Supplementary Table 3** Primers used in this study.

| **Gene Name** | **Accession No.** | **Sequence (5'-3')** | **Size (bp)** |
| --- | --- | --- | --- |
| GAD1 | NM_213894.1 | GAGCGAACAGGTTGGAGAAG AGAAGAGCAGGCTTGTGAGC | 126 |
| GAD2 | NM_213895.2 | AGACAAGGCCTTACAGTGCG TGGCCTTAATCACTGGAGCC | 195 |
| SLC6A12 | XM_003126574.4 | GATGGAGTTCGTGCTGTCTG CCGCAGGTGAAGAAGAAGAT | 132 |
| SLC6A11 | XM_005657080.1 | GGGCATTCCTGATTCCCTAT GGCACACTTTCCTCCAACAC | 123 |
| SLC6A13 | XM_003126576.4 | CAGTTTGTGTGCGTGGAAAG ACGAGGAAGGAGGTGACAGA | 119 |
| SLC6A1 | XM_005669778.2 | CTGGACTGGAAAGGTGGTCT GCGGAAGTTGGGTGTGATA | 130 |
| GABA_B_1 | NM_001123114.1 | GCCCGTGGACTATGAGATTG CTTGGAGCAGATTCGGACAC | 136 |
| GABA_B_2 | XM_003122032 | CATCACCCTTTGCCTGGTGT AGGTGGTCTTTTCTGGTGTGT | 275 |
| GABA_A_α1 | XM_013984876 | GCCTTCCCATTGCTGTTTGG GCAGAGGACGGAACAACAGA | 132 |
| GABA_A_α2 | XM_013978644 | GGATGATGGGACTCTGCTGT TCAGAGGACACGAGTGAGCA | 101 |
| GABA_A_α3 | XM_003135469 | TGGCCAAGGGGAATCAAGAC TCGGTCACTGCATCTCCAAG | 195 |
| GABA_A_α4 | XM_003356906 | ACCTCAGACGGAAGATGGGT GCTCAGTGTGGTCATGGTGA | 158 |
| GABA_A_α5 | XM_003121648 | GGCTAAACCGAGAATCAGTCC ACACGGCTATGAACCAGTCC | 141 |
| GABA_A_β2 | XM_013984877 | GCTGCTAGTGCCAACAATGA GTTGGGGAGAGGTTTCCAGT | 122 |
| GABA_A_δ | XM_013988717 | TCTCCTACAACCACACCAACG AGCCTGATGAGCTTGTTCTCC | 139 |
| GABA_A_ε | XM_013986487 | ACCTGGTGAGCCTGTTATGG GTACAACACCTTGCCGTCCT | 122 |
| GABA_A_γ1 | XM_003128952.5 | CAAAACTTGGGTCTTGGCACC TACTGTGGGCCTCACTCCTA | 118 |
| GABA_A_γ2 | XM_003359825 | CTCCCAATAGGATGCTGAGG GGCAGGAGTGTTCATCCATT | 120 |
| GABA_A_π | XM_003134060.4 | CTTCACTTTGGATGCACGCC ACAGGACTGTGCCATTGGAG | 137 |
| GABA_A_θ | XM_013986506 | AGAGGCAACTCACAAGAGCC GGGGGACTTTCCTCATCAGC | 151 |
| GABA_A_ρ1 | XM_013992689.1 | AACGACCTGGGCAACTACAC TGCTCCTCTGGCTCTTCCT | 112 |
| TLR2 | NM_213761.1 | TGCTATGACGCTTTCGTGTC CGATGGAGTCGATGATGTTG | 163 |
| TLR4 | XM_013986843.1 | TCAGTTCTCACCTTCCTCCTG GTTCATTCCTCACCCAGTCTTC | 166 |
| TLR5 | NM_001123202.1 | CCTTCCTGCTTCTTTGATGG CTGTGACCGTCCTGATGTAG | 124 |
| TLR6 | NM_213760.1 | AACCTACTGTCATAAGCCTTCATTC GTCTACCACAAATTCACTTTCTTCAG | 95 |
| MyD88 | XM_013992286.1 | GATGGTAGCGGTTGTCTCTGAT GATGCTGGGGAACTCTTTCTTC | 148 |
| IL-1 | NM_001302388.1 | AAAGGGGACTTGAAGAGAG CTGCTTGAGAGGTGCTGATGT | 286 |
| IL-18 | NM_213997.1 | TATGCCTGATTCTGACTGTT ATGAAGACTCAAACTGTATCT | 260 |
| TNF-α | NM_214022.1 | TTCCTCACTCACACCATCAGCC TGCCCAGATTCAGCAAAGTCC | 224 |
| IL-2 | NM_213861.1 | CTGGATTTACAGTTGCTTTTG AGTCAGTGTTGAGTAGATGCTTT | 348 |
| IFN-γ | NM_213948.1 | GGCCATTCAAAGGAGCATGG GATGGCTTTGCGCTGGATCT | 139 |
| IL-10 | NM_214041.1 | CATCCACTTCCCAACCAGCC CTCCCCATCACTCTCTGCCTTC | 220 |
| IL-6 | NM_001252429.1 | TGGCTACTGCCTTCCCTACC CAGAGATTTTGCCGAGGATG | 132 |
| IL-4 | NM_214123.1 | TCCACGGACACAAGTGCGAC TGTTTGCCATGCTGCTCAGG | 243 |
| IL-17 | NM_001005729.1 | ATCCTCGTCCCTGTCACTGC  ACATGCTGAGGGAAGTTCTTGTC | 120 |
| IL-22 | XM_001926156.1 | GATGAGAGAGCGCTGCTACCTGG GAAGGACGCCACCTCCTGCATGT | 112 |
| ACTB | XM_003124280.4 | CTGCGGCATCCACGAAACT AGGGCCGTGATCTCCTTCTG | 147 |
| GAPDH | NM_001206359.1 | ATCCTGGGCTACACTGAGGAC AAGTGGTCGTTGAGGGCAATG | 104 |

Primers were designed under the principle of primer design with Primer 6.0 software (PRIMERE, New Zealand) and Oligo 5.0 software (Molecular Biology Insights, Inc., USA) according to the gene sequences of sus scrofa on GenBank.

Supplementary Table 4 Alpha diversity indices of the ileal microbiota in piglets.

|  | Control | *L.lactis* | *P*-value |
| --- | --- | --- | --- |
| chao1 | 193.13±27.56 | 216.96±31.58 | 0.22 |
| ace | 198.94±27.26 | 223.49±32.08 | 0.15 |
| shannon | 2.08±0.24 | 2.69±0.35 | 0.61 |
| simpson | 0.52±0.05 | 0.65±0.06 | 0.60 |

Richness estimator (Chao1 and AEC) and diversity estimator (Shannon, Simpson) were statistically calculated by software named mothur. Piglets from control group (n=7) and *L.lactis* group (n=8). Both unpaired t-test and Wilcoxon nonparametric tests were used for the analysis of the data (mean ± SEM).
